# Prescriptions and Dispensing

**Published:** 1908-06-13

**Authors:** 


### THERAPEUTICS.

### PRESCRIPTIONS AND DISPENSING.

### PILLS (*continued.*)

It may be desired to prescribe quite out-of-the-way powders in pill form, and it may not at first be quite obvious how to make them up; but although powders may differ much in composition and therapeutic action, their physical properties usually bring them under some familiar class; by considering whether a powder is soluble in water or spirit, whether it contains vegetable tissue or not, whether it is a single chemical substance or a complex mixture, and a few other such questions, it is usually not difficult to succeed in massing it with the first excipient that suggests itself as likely to be a good one for the occasion.

It often happens that the ingredients required in the pill may be such that the question is, not how to bring dry powders into the form of a coherent mass, but how to bring a mixture of soft extracts, or even a liquid, to a sufficiently solid state to make it into pills.

There are two principal ways of doing this: one is to add a sufficient quantity of a dry powder or other absorbent to stiffen the mass; the other is to remove the superfluous moisture by evaporation. Very commonly both methods need to be employed. The two powders chiefly used to absorb moisture and stiffen a mass are liquorice root and marsh-mallow root, each in very fine powder. Sometimes the further addition of a small quantity of tragacanth is necessary.

Such pills as the following—

℞ Extracti Belladonnæ liquidi ... ... gr. $\frac{1}{6}$

Extracti Hyoscyami ... ... ... gr. $\frac{1}{4}$

Extracti Opii ... ... ... ... gr. $\frac{1}{2}$

Fiat pilula j.

℞ Extracti Colchici ... ... ... ... gr. $\frac{1}{2}$

Extracti Nucis Vomicæ ... ... ... gr. $\frac{1}{4}$

Fiat pilula j.

will mass quite easily with the addition of a little liquorice, and this method should be employed; but with such as the following—

℞ Extracti Ergotæ ... ... ... ... gr. XL.
Fiant pilulæ viij.

℞ Elaterini ... ... ... ... ... gr. $\frac{1}{10}$

Extracti Hyoscyami ... ... ... gr. iv.

Fiat pilula j.

it is at once evident that so much powder would be required that the pills would be of excessive size. In these cases evaporation must be employed. The ingredients are placed in a small flat-bottomed dish, or on a glazed tile, and the dish or tile heated by means of a water-bath. It is a safe rule always to heat extracts or other vegetable preparations as little as possible, and the mass should accordingly be worked with a pill-knife during the heating, as evaporation is thus promoted and the time of evaporation reduced. Pills like the above, which consist entirely, or almost entirely, of soft extracts, require the addition of about one grain of liquorice root powder to each pill, in addition to removing moisture. It must be remembered that the mass will be considerably stiffer when cold than while still hot, so that it may be removed from the water-bath whilst still fairly soft; it should then be rolled and finished without loss of time. The finished pills should be weighed, and a note of the weight kept, for future guidance, so that the patient may receive the same size of pill should he wish the medicine repeated as before.

It is a convenient plan to keep some of the commoner extracts in a ready-dried condition. Liquorice powder is mixed with them in sufficient quantity to make up to the original weight, or to a known and convenient weight less than the original; in the latter case the ratio between equivalent quantities of the soft and dried extract must be plainly noted on the label; as, for instance, "Ext. ergotæ sicc., 4 grains equals 5 grains soft extract." Some extracts can be brought to a powder in this way, but the form of granules is more easily attained, and it is very convenient.

*Essential oils* often form ingredients in pills, and they are occasionally the principal ingredients; in such cases it is, of course, necessary to employ some substance that will absorb the oil, as any evaporation would here remove, not moisture only, but the actual medicament itself. Soap is usually the best thing for making essential oils into pills. If a dry vegetable powder is not being prescribed, some liquorice should also be added; generally, about $\frac{1}{2}$ grain of soap and 2 to 3 grains of liquorice will suffice for 1 minim of essential oil; spirit or water will then usually suffice for massing. Curd soap is better in most cases than Castile.

*Creosote* is often ordered in pills, and may be made up by the method just given for essential oils; for mixing with other ingredients it is a good plan to put equal parts of curd soap and creosote into a wide-mouthed stoppered bottle, and heat in a water-bath till they amalgamate; on cooling, a mass is obtained which is well suited for mixing with other substances to make pills. A small creosote pill may be made by taking $\frac{1}{2}$ grain of curd soap for each drop of creosote, and adding enough calcium phosphate to stiffen into a mass.

*Croton oil*, though not often ordered now, may be given in pill form; in this case also curd soap is a most useful addition; a little glycerin of tragacanth may be needed for massing.

*Pills Containing Ingredients that React Together.*

Chemical reaction between the ingredients of a pill does not take place as easily as it does between the constituents of a liquid mixture; nevertheless, it cannot be ignored. In certain cases it may be intended that double decomposition shall take place, but in others such action may not have been foreseen. One of the best-known instances in which mutual reaction is intended is Blaud's pill, the Pilula Ferri of the Pharmacopœia. Blaud's pill is intended for the administration of ferrous carbonate. This substance is rapidly decomposed if open to the air, the principal product being ferric oxide; it is therefore necessary that the pills taken by the patient should be fresh. Dried sulphate of iron is mixed with glycerin, water, and syrup, and dried sodium carbonate is added. The water dissolves a portion of each salt, these at once reacting together to form sodium sulphate and insoluble ferrous carbonate. Further quantities of ferrous sulphate and sodium carbonate are then dissolved, and react in the same way, so that after a few minutes the whole of the mixed salts become converted into ferrous carbonate and sodium sulphate. Meanwhile, the glycerin and sugar protect the ferrous carbonate from being actively oxidised; in the pharmacopœial directions, fifteen minutes' standing is ordered for the completion of the reaction; acacia and a little tragacanth are then added, and the whole worked into a mass. When the pills are dry, the ferrous carbonate is out of contact with the air except on the extreme surface, and will keep unchanged for a long time; even the surface action is considerably delayed if the pills be coated.

*(To be continued.)*

